# OASL1 deficiency promotes antiviral protection against genital herpes simplex virus type 2 infection by enhancing type I interferon production

**DOI:** 10.1038/srep19089

**Published:** 2016-01-11

**Authors:** Ji Eun Oh, Myeong Sup Lee, Young-Joon Kim, Heung Kyu Lee

**Affiliations:** 1 Laboratory of Host Defenses, Graduate School of Medical Science and Engineering, Korea Advanced Institute of Science and Technology (KAIST), Daejeon, 34141, Republic of Korea; 2 Department of Biomedical Sciences, College of Medicine, University of Ulsan, Seoul, 05505, Republic of Korea; 3 Department of Biochemistry, College of Life Science and Technology, Yonsei University, Seoul, 03722, Republic of Korea; 4 Department of Integrated Omics for Biomedical Science Graduate School, Yonsei University, Seoul, 03722, Republic of Korea

## Abstract

Type I interferon (IFN) interferes with virus replication, promotes antiviral responses, and controls innate and adaptive immune responses to certain viruses. Recently, we reported that 2’–5’ oligoadenylate synthetase-like 1 (OASL1) negatively regulates type I IFN production by inhibiting the translation of the type I IFN-regulating master transcription factor, IRF7. Notably, while OASL1-deficient mice induce robust production of type I IFN and are resistant to systemic viral infection, the effects of OASL1 during localized viral infection has not been studied. To this end, we investigated the role of OASL1 during mucosal HSV-2 infection of the genital tract. *Oasl1*^−/−^ mice exhibited better survival rates than wild type (WT) mice following intravaginal HSV-2 infection, and suppressed virus replication more efficiently despite comparable recruitment of effector immune cells. Moreover, Ly6C^high^ monocytes, and not pDCs or other cell types, displayed enhanced production of type I IFNs in *Oasl1*^−/−^ mice in response to HSV-2 infection. Furthermore, cytotoxic T cell responses including IFN-γ production were accelerated in *Oasl1*^−/−^ mice after mucosal HSV-2 infection. Collectively, these results demonstrate that OASL1 deficiency promotes antiviral immunity against local mucosal viral infection and suggest that OASL1 could be a therapeutic target for treatment of HSV-2 infection of the genital mucosa.

Genital herpes is one of the most common sexually transmitted infections (STIs) worldwide. It is caused by herpes simplex virus type 2 (HSV-2), which has a linear, double-stranded DNA genome of ~154 kb in length. HSV-2 infection not only causes ulcers within the genital tract, but also induces lifelong latency within the sensory ganglia of the nervous system. In addition to genital ulcers, HSV-2 infection can cause severe and frequently fatal symptoms, and is considered to be a major risk factor for other STIs such as human immunodeficiency virus type 1 (HIV-1)^1^. Although the prevalence of HSV-2 infection and the incidence of genital herpes have been increasing, there is currently a lack of effective therapeutics^2^. Thus, understanding host immune responses against HSV-2 infection may provide clues for the cure and prevention of this debilitating disease.

The innate immune system represents the first line of defense against pathogens and acts by limiting infection or replication and by initiating the adaptive immune response. Notably, type I IFNs are critical for inhibition of early viral replication, activation of immune cells, and regulation of adaptive immune responses. In this regard, after genital infection with HSV-2, IFNα/β receptor-deficient (IFNAR^−/−^) mice showed increased viral replication and decreased survival rates compared to wild type (WT) mice^3^.

Type I IFNs induce various interferon-stimulated genes (ISGs), which are involved in diverse antiviral pathways^4^,^5^. Collectively, ISGs inhibit viral protein synthesis and virus replication, thus providing early protection against virus infection. For example, protein kinase R (PKR), the dsRNA-activated serine/threonine protein kinase, is an ISG that negatively regulates mRNA translation. Other ISGs, such as 2′–5′-oligoadenylate synthetase (OAS) and RNase L, are also involved in the degradation of both cellular and viral RNA. Moreover, type I IFNs activate innate immune cells including natural killer (NK) cells, which then lyse virus-infected cells, and dendritic cells (DCs), inducing their maturation through expression of MHC and co-stimulatory molecules^6^. Interestingly, type I IFNs can also activate and expand antigen-specific T cells^6^. Thus, type I IFNs regulate adaptive immune responses both directly and indirectly.

Type III IFNs, comprised of IFN-λ1, -λ2, and -λ3, are a newly identified subset of IFNs^7^,^8^. Although type III IFNs signal through distinct receptor complexes from type I IFNs, the biologic functions and downstream signaling pathways are similar^9^. What makes type III IFNs unique is the restriction of their receptors to epithelial tissue^10^. Moreover, recent studies demonstrated that IFN-λ plays critical roles in the antiviral protection of the mucosal organ^11^,^12^. In the case of mucosal HSV-2 infection, IFN-λ has been shown to inhibit virus replication in the vaginal mucosa thereby conferring protection against HSV-2 infection^13^,^14^.

Recently, we showed that OASL1, a nonenzymatic OAS protein, negatively regulates the production of type I IFNs during viral infection by inhibiting the translation of interferon regulatory factor 7 (IRF7)^15^. Following virus recognition by various receptors, the production of type I IFNs is induced through activation of IRF3^16^. IRF3 is the key transcription factor leading to the early production of type I IFNs (predominately of IFN-β), which initiates a positive feedback loop in autocrine and paracrine manners^17^,^18^. In this process, IRF7, a master regulator of type I IFNs, functions to further amplify the expression of type I IFNs^19^. Thus, *Oasl1*^−/−^ mice are resistant to systemic viral infection due to increased production of type I IFNs^15^. In addition, another study using a systemic chronic lymphocytic choriomeningitis virus (LCMV) infection model demonstrated that OASL1-mediated suppression of type I IFN production prevents efficient viral control and the induction of a functional T cell response, permitting viral persistence^20^. Notably, the function of OASL1 in a non-systemic, natural mucosal virus infection remains unknown. Furthermore, whether OASL1 also regulates type III IFNs has not been investigated.

In the present study, we show that *Oasl1*^−/−^ mice are more resistant to mucosal HSV-2 infection as compared to WT mice. Furthermore, hematopoietic cells were sufficient for this enhanced protection of *Oasl1*^−/−^ mice against mucosal HSV-2 infection. Although production of type III IFNs was not increased in *Oasl1*^−/−^ BM cells after *in vitro* stimulation with HSV-2, type III IFN remained high in vaginal washes until later time points after intravaginal HSV-2 infection. The increased production of type I IFNs in *Oasl1*^−/−^ mice was derived from Ly6C^high^ monocytes, not from plasmacytoid DCs (pDCs), and effectively induced robust CD8^+^ T cell responses protecting against mucosal HSV-2 infection. Together, these results indicate that OASL1-mediated negative regulation of type I IFN production suppresses both innate and adaptive immunity against mucosal HSV-2 infection.

## Results

### *Oasl1* ^−/−^ mice are more resistant to mucosal HSV-2 infection than WT mice

**Figure 1:**
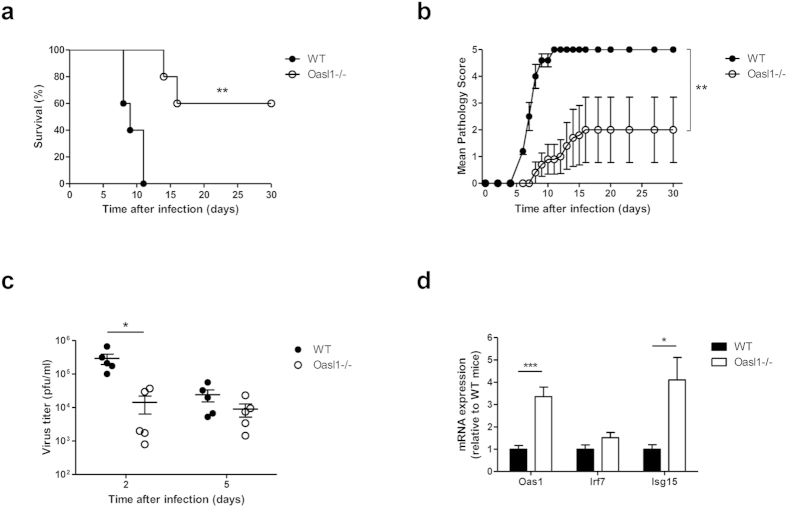
OASL1 deficiency enhances immune protection against mucosal HSV-2 infection. WT and *Oasl1*^−/−^ mice were infected intravaginally with 1000 pfu of WT HSV-2. (**a**) Survival and (**b**) disease scores were monitored for one month post-challenge (n = 5 mice per group). Data are representative of four independent experiments. (**c**) At the indicated days post infection, HSV-2 viral titers from vaginal washes were measured on Vero cells (n = 5 mice). Data are representative of three independent experiments. (**d**) Expression of ISGs in vaginal tissue of uninfected *Oasl1*^−/−^ mice relative to that of uninfected *Oasl1*^+/−^ mice was determined by qRT-PCR (n = 5 mice). Data are representative of two independent experiments. *p < 0.05; **p < 0.01; ***p < 0.001. Error bars: SEM.

In our previous study, we found that OASL1 inhibited the translation of IRF7, a master transcription factor for type I IFN production^15^. Thus, OASL1 negatively regulates excessive production of type I IFN to limit hyperinflammatory responses. In this regard, *Oasl1*^−/−^ mice produce more type I IFN after poly (I:C) treatment and are more resistant to systemic virus infection than WT mice^15^. To determine whether *Oasl1*^−/−^ mice are also more resistant to local mucosal virus infection, we infected *Oasl1*^−/−^ and littermate WT control mice intravaginally with 1000 pfu of WT HSV-2. Only two-fifths of *Oasl1*^−/−^ mice died after genital HSV-2 infection, while all WT mice died within 11 days of infection (Fig. 1a). Moreover, *Oasl1*^−/−^ mice showed only mild clinical pathology (Fig. 1b), and viral titers from vaginal washes were markedly lower in *Oasl1*^−/−^ mice at early time points post-infection as compared to WT mice (Fig. 1c). Interestingly, we found that ISGs such as OAS1 and ISG15 were markedly increased in vaginal tissue of *Oasl1*^−/−^ mice even in the absence of infection (Fig. 1d). This indicates that the enhanced antiviral state within the vaginal tract limited viral replication early after infection in *Oasl1*^−/−^ mice and that these mice are more resistant to local mucosal HSV-2 infection than are WT mice.

### *Oasl1* ^−/−^ hematopoietic cells are sufficient for enhanced protection against mucosal HSV-2 infection

**Figure 2:**
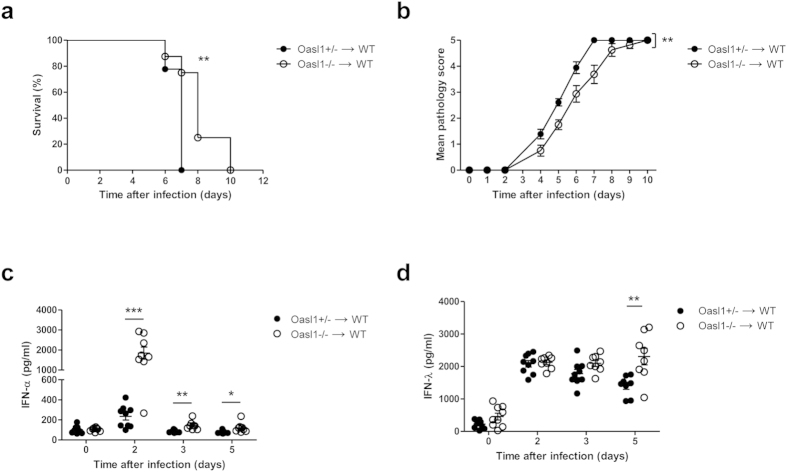
Hematopoietic cells contribute to enhanced immune protection against mucosal HSV-2 infection in *Oasl1*^−/−^ mice. *Oasl1*^+/−^→WT and *Oasl1*^−/−^→WT chimera mice were infected intravaginally with 10000 pfu of WT HSV-2. (**a**) Survival and (**b**) disease scores were monitored until all mice succumbed. (**c,d**) On the indicated days post infection, vaginal washes were collected and the level of (**c**) IFN-α and (**d**) IFN-λ was measured by ELISA (*Oasl1*^+/−^→WT, n = 9 mice; *Oasl1*^−/−^→WT, n = 8 mice). *p < 0.05; **p < 0.01; ***p < 0.001. Error bars: SEM.

Unlike systemic viral infection, both hematopoietic and stromal compartments take part in innate immune responses after mucosal HSV-2 infection. Mucosal epithelial cells are the first cell types infected with HSV-2 that produce type I IFN, albeit much less than hematopoietic cells. These type I IFNs, primarily IFN-β, initiate a positive feedback loop, thus promoting robust production of more type I IFN by hematopoietic cells recruited to the site of infection. In this regard, Shen and Iwasaki reported that mice lacking IFNαβR expression on hematopoietic cells displayed a more severe phenotype in response to mucosal HSV-2 infection than mice lacking IFNαβR on stromal cells^21^. To elucidate whether *Oasl1*^−/−^ hematopoietic cells are sufficient for the enhanced protection against mucosal HSV-2 infection, we generated irradiation-induced BM chimera mice that lack OASL1 expression in hematopoietic cells but have intact OASL1 expression in stromal cells. Compared with *Oasl1*^+/−^→WT mice, *Oasl1*^−/−^→WT mice survived longer and showed milder disease pathology following intravaginal infection with WT HSV-2 (Fig. 2a,b). Interestingly, *Oasl1*^−/−^→WT mice produced a profuse amount of IFN-α at the infection site two days post-infection (Fig. 2c).

The recently identified type III IFN, IFN-λ, has been shown to confer antiviral protection to the mucosal epithelia^10^,^11^. Type III IFNs bind different receptors than type I IFNs, but induce the same signaling pathways^9^. Thus, to determine whether OASL1 also regulates type III IFN, we measured the level of IFN-λ in vaginal washes after mucosal HSV-2 infection. Unlike IFN-α, the level of IFN-λ at the infection site in *Oasl1*^−/−^→WT mice was comparable to *Oasl1*^+/−^→WT mice at early time points post-infection. However, high levels of IFN-λ in *Oasl1*^−/−^→WT mice were prolonged until late time points post-infection, while IFN-λ levels in *Oasl1*^+/−^→WT mice gradually decreased (Fig. 2d). Taken together, these data suggest that hematopoietic cells are sufficient for protection against mucosal HSV-2 infection in *Oasl1*^−/−^ mice through the enhanced production of type I and type III IFNs.

### Production of type I IFNs, but not type III IFNs and proinflammatory cytokines, is enhanced in *Oasl1* ^−/−^ bone marrow cells

**Figure 3:**
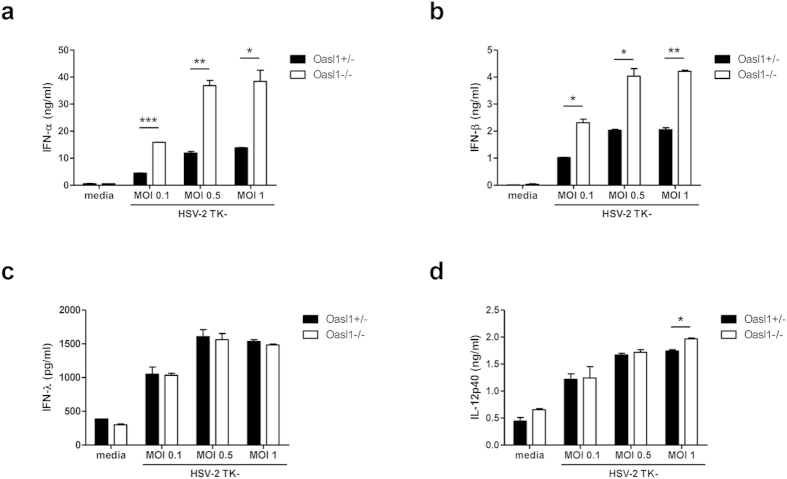
*Oasl1*^−/−^ BM cells produce more type I IFNs, but not IFN-λ and IL-12p40. BM cells from *Oasl1*^+/−^ and *Oasl1*^−/−^ mice were stimulated with TK- HSV-2 at the indicated MOIs for 18 h. Levels of (**a**) IFN-α, (**b**) IFN-β, (**c**) IFN-λ, and (**d**) IL-12p40 in supernatants were measured by ELISA (n = 2). Data are representative of two to three independent experiments. *p < 0.05; **p < 0.01; ***p < 0.001. Error bars: SEM.

Based on the above results, we next wanted to examine the role of hematopoietic cells in *Oasl1*^−/−^ mice in response to HSV-2 infection. To this end, we treated bone marrow (BM) cells with TK- HSV-2 at various multiplicities of infection (MOI) *in vitro*. We found that *Oasl1*^−/−^ BM cells produced significantly more type I IFN, including IFN-α and IFN-β, when stimulated with HSV-2 (Fig. 3a,b). However, IFN-λ production by *Oasl1*^−/−^ BM cells was comparable to that of *Oasl1*^+/−^ BM cells after stimulation with HSV-2 (Fig. 3c). In addition, similar amounts of IL-12p40, a pro-inflammatory cytokine important for the differentiation of Th1 cells, were produced by infected control and *Oasl1*^−/−^ BM cells (Fig. 3d). These data indicate that OASL1 selectively suppresses the production of type I IFN, but not type III IFN or pro-inflammatory cytokines after HSV-2 infection.

### Ly6C^high^ monocytes are major sources of enhanced production of type I IFNs in *Oasl1* ^−/−^ mice in response to HSV-2 infection

**Figure 4:**
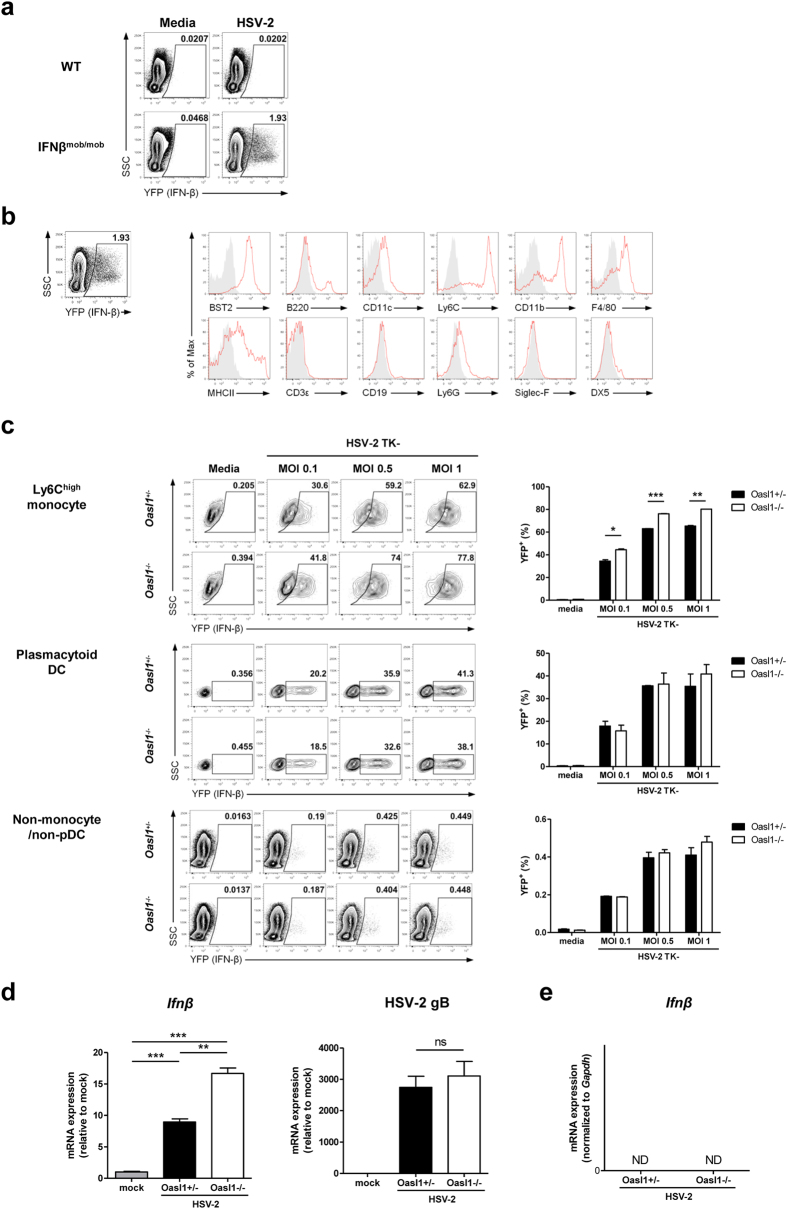
Ly6C^high^ monocytes are major contributors of the enhanced production of type I IFNs in *Oasl1*^−/−^ mice after infection with HSV-2. (**a,b**) BM cells from WT and IFNβ^mob/mob^ mice were stimulated with TK- HSV-2 (MOI 5) for 18 h. (**a**) YFP expression was assessed using flow cytometry. Plots were gated on PI^-^ cells. (**b**) Surface expression of indicated molecules (red histograms) on YFP^+^ cells from BM of IFNβ^mob/mob^ mice was analyzed by flow cytometry. Shaded gray histograms indicate isotype control. (**c**) BM cells from *Oasl1*-heterozygous IFNβ^mob/mob^ and *Oasl1*-deficient IFNβ^mob/mob^ mice were stimulated with TK- HSV-2 at the indicated MOIs for 18 h. **left**. YFP expression in Ly6C^high^ monocytes, pDCs, and non-monocytes/non-pDCs was assessed using flow cytometry. Numbers indicate the percentage of cells with YFP expression. **right**. Bar graphs show the percentage of YFP expression shown in left panels (n = 2). Data are representative of three independent experiments. (**d**) Expression of *Ifnβ* and HSV-2 gB in sorted Ly6C^high^ monocytes from vaginal tissue of intravaginal WT HSV-2 infected *Oasl1*^+/−^ and *Oasl1*^−/−^ mice relative to sorted Ly6C^high^ monocytes from BM cells of uninfected WT mice (mock) was determined by qRT-PCR (n = 6 mice pooled per group). (**e**) Expression of *Ifnβ* in sorted CD4^+^ T cells from vaginal tissue of intravaginal WT HSV-2 infected *Oasl1*^+/−^ and *Oasl1*^−/−^ mice was determined by qRT-PCR. Data are representative of two independent experiments. *p < 0.05; **p < 0.01; ***p < 0.001; ns, not significant. Error bars: SEM.

Although most types of cells can produce type I IFN, certain cells such as pDCs robustly produce type I IFN in response to viral infection. In the case of HSV infection, it has been reported that pDCs are indispensable for early antiviral protection due to their ability to produce type I IFN^22^. However, a recent study using transgenic mice selectively depleting pDCs showed that pDCs are critical for antiviral immunity against systemic HSV, but not local HSV infection^23^. In addition, this study suggested that pDCs are not the only source of IFN-α during systemic HSV infection. To elucidate what is the primary source of type I IFN during HSV-2 infection, we infected BM cells isolated from IFNβ^mob/mob^ mice, which express yellow fluorescent protein (YFP) in an IFN-β-dependent manner, with HSV-2 *in vitro*. First, we confirmed that YFP was expressed specifically in this reporter mouse after infection with HSV-2 (Fig. 4a). Next, we investigated the surface phenotype of YFP^+^/IFN-β-producing cells in BM cells stimulated with HSV-2 in order to determine the cell type producing type I IFN following HSV-2 infection. Strikingly, while YFP^+^/IFN-β-producing cells were positive for surface markers of pDCs such as BST2, B220, and CD11c, some molecules not expressed by pDCs, such as CD11b, were also expressed on YFP^+^/IFN-β-producing cells. In this regard, we found that YFP^+^/IFN-β-producing cells also expressed high levels of Ly6C and F4/80, and intermediate levels of Ly6G and not Siglec-F, suggesting that these cells are Ly6C^high^ monocytes (Fig. 4b).

As shown in Fig. 3, *Oasl1*^−/−^ cells produced more type I IFN after infection with HSV-2. To determine whether enhanced production of type I IFN in *Oasl1*^−/−^ cells is cell-type dependent, we examined the expression of YFP in BM cells from *Oasl1*-heterozygous IFNβ^mob/mob^ mice or *Oasl1*-deficient IFNβ^mob/mob^ mice after infection with HSV-2. YFP expression was induced by both Ly6C^high^ monocytes and pDCs after stimulation with HSV-2 in MOI-dependent manner. We detected greater YFP expression in Ly6C^high^ monocytes, but not in pDCs or other cells, in *Oasl1*^−/−^ mice than in control mice after HSV-2 infection (Fig. 4c and supplementary Fig. 1). In addition, to examine whether Ly6C^high^ monocytes became directly infected by HSV-2 and then produced type I IFN or if Ly6C^high^ monocytes produced type I IFN in a paracrine manner, we examined the expression of GFP in BM monocytes after infection with GFP HSV-1. We found that GFP expression in BM monocytes increased in an MOI-dependent manner, and the level of GFP expression was not different between *Oasl1*^+/−^ and *Oasl1*^−/−^ BM monocytes (Supplementary Fig. 2).

Next, to investigate whether Ly6C^high^ monocytes infiltrated in vaginal tissues contributed to the enhanced production of type I IFN in *Oasl1*^−/−^ mice *in vivo*, we compared IFN-β mRNA expression in Ly6C^high^ monocytes sorted from infected vaginal tissues with BM monocytes (mock control) (Fig. 4d and supplementary Fig. 3). Interestingly, we detected increased IFN-β mRNA expression in vaginal Ly6C^high^ monocytes following HSV-2 infection, whereas IFN-β expression was not detected in vaginal CD4^+^ T cells (Fig. 4d,e). Further, *Oasl1*^−/−^ mice showed higher expression of IFN-β in vaginal Ly6C^high^ monocytes than did *Oasl1*^+/−^ mice (Fig. 4d). Furthermore, similar levels of HSV-2 glycoprotein B (gB) were detected in Ly6C^high^ monocytes sorted from vaginal tissues of genital HSV-2 infected *Oasl1*^+/−^ and *Oasl1*^−/−^ mice (Fig. 4d).

Taken together, our data indicate that the major cell type contributing to the robust production of IFN-β in *Oasl1*^−/−^ mice are Ly6C^high^ monocytes *in vitro* and *in vivo*, although substantial amounts of IFN-β can be produced by both Ly6C^high^ monocytes and pDCs in response to HSV-2 infection. Moreover, Ly6C^high^ monocytes from *Oasl1*^−/−^ mice produce higher levels of type I IFNs than those from *Oasl1*^+/−^ mice, even though these cells are infected by HSV-2 at similar levels.

### Recruitment of innate immune cells in *Oasl1* ^−/−^ and control mice is comparable

**Figure 5:**
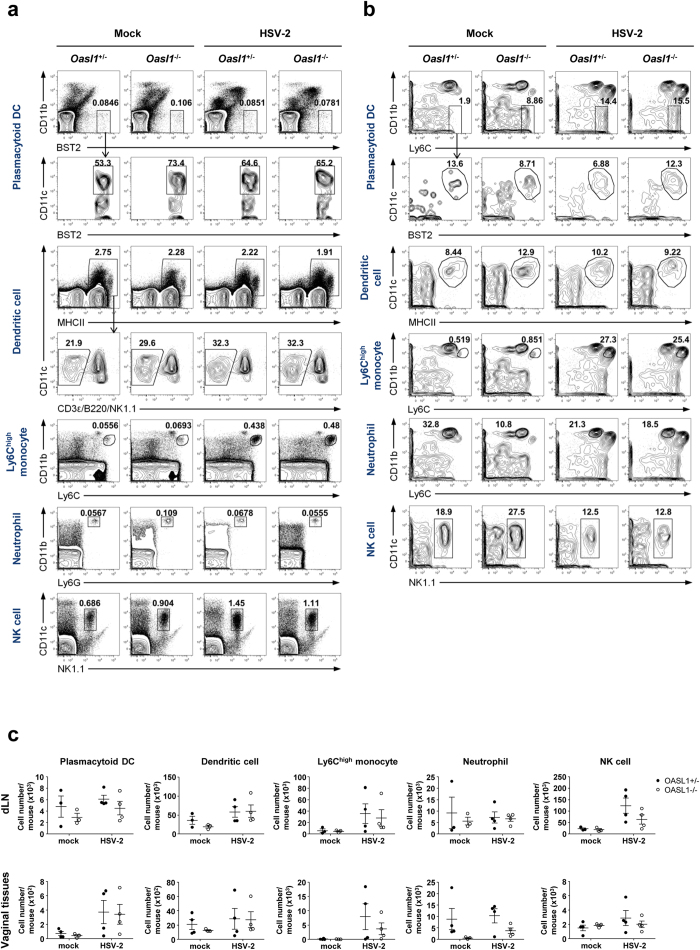
Innate immune cells migrate normally into the site of mucosal HSV-2 infection of *Oasl1*^−/−^ mice. *Oasl1*^+/−^ and *Oasl1*^−/−^ mice were infected intravaginally with 500 pfu of WT HSV-2. At day 3 post-infection, (**a**) iliac lymph nodes and (**b**) vaginal tissues were collected, and the frequency of immune cells was analyzed by flow cytometry. Plots were gated on PI^-^ (iliac LN) and PI^-^CD45.2^+^ (vaginal) cells. Numbers indicate the percentage of gated cells. Results are representative of two experiments with 3–4 mice per group. (**c**) Number of innate immune cells in iliac lymph nodes and vaginal tissues was assessed using flow cytometric analysis (n = 3–4 mice per group). Data are a compilation of two experiments. Error bars: SEM.

After viral infection, innate effector cells migrate to the site of infection to defeat the infection through cytokine production and killing of viral-infected cells. To investigate the possibility that OASL1 deficiency affects the trafficking of innate immune cells to the site of infection, we examined the proportion and the number of effector immune cell subsets, including pDCs, DCs, Ly6C^high^ monocytes, neutrophils, and NK cells, in draining lymph nodes and vaginal tissues early after infection (Fig. 5). We found that there was no substantial difference between *Oasl1*^+/−^ and *Oasl1*^−/−^ mice in the proportion or number of innate immune cells in either draining lymph nodes or vaginal tissues. Taken together, these results suggest that migration of innate effector cells is not affected by the absence of OASL1 protein.

### Expression of co-stimulatory and MHC molecules on antigen presenting cells is similar between *Oasl1* ^−/−^ and control mice

**Figure 6:**
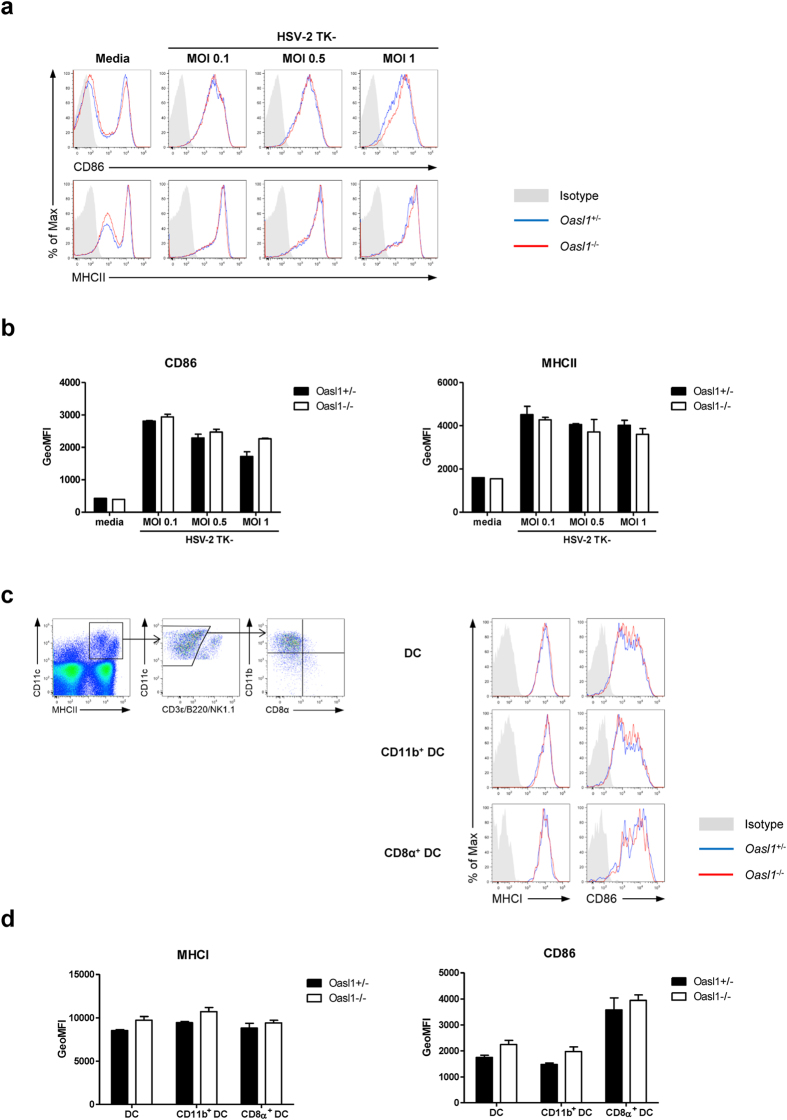
Expression of co-stimulatory molecules on antigen presenting cells in *Oasl1*^−/−^ mice is comparable to that in control mice. (**a,b**) BM-derived dendritic cells (BM-DCs) generated from *Oasl1*^+/−^ and *Oasl1*^−/−^ mice were stimulated with TK- HSV-2 at the indicated MOIs for 18 h. (**a**) Surface expression of CD86 and MHCII on CD11c^+^ cells from BM-DCs of *Oasl1*^+/−^ (blue histograms) and *Oasl1*^−/−^ (red histograms) mice was analyzed by flow cytometry. Shaded gray histograms indicate isotype control. (**b**) Geometric mean fluorescent intensity (GeoMFI) of surface expression of CD86 and MHCII molecules shown in (**a**) (n = 2). Data are representative of two independent experiments. (**c,d**) *Oasl1*^+/−^ and *Oasl1*^−/−^ mice were infected intravaginally with 10^7^ pfu of TK- HSV-2. At day 3 post-infection, iliac lymph nodes were collected. (**c**) ***left.*** Gating strategies of DCs and DC subsets. ***right.*** Surface expression of MHCI and CD86 on the indicated cell types from draining lymph nodes of infected *Oasl1*^+/−^ (blue histograms) and *Oasl1*^−/−^ (red histograms) mice were analyzed by flow cytometry. Shaded gray histograms indicate isotype control. DC was defined as MHCII^+^CD11c^+^CD3ε^-^B220^-^NK1.1^-^ cells, CD11b^+^ DC as CD11b^+^CD8α^-^ DC, and CD8α^+^ DC as CD8α^+^CD11b^-^ DC. (**d**) GeoMFI of surface expression of MHCI and CD86 molecules shown in (**c**) (n = 2). Data are representative of two independent experiments. Error bars: SEM.

Type I IFN modulates various immune cell functions including the development, maturation, migration, and antigen presentation of antigen presenting cells (APC), the most important mediators of innate and adaptive immunity^6^. To investigate whether the increased production of type I IFN in *Oasl1*^−/−^ mice affects DC maturation, we measured the expression of co-stimulatory and MHC molecules on DCs following HSV-2 infection. To this end, BM-derived DCs from *Oasl1*^−/−^ mice showed comparable expression of CD86 and MHCII to DCs from control mice after stimulation with HSV-2 *in vitro* (Fig. 6a,b). Moreover, the level of expression of CD86 and MHCI on various DC subsets including CD11b^+^ DCs and CD8α^+^ DCs was not significantly different between *Oasl1*^−/−^ and control mice three days after mucosal HSV-2 infection (Fig. 6c,d). Collectively, DC maturation was not enhanced in *Oasl1*^−/−^ mice, even though production of type I IFNs was greatly enhanced in *Oasl1*^−/−^ mice in response to HSV-2 infection. These results suggest that the amount of type I IFN produced by control mice might be sufficient for inducing DC maturation after HSV-2 infection.

### Augmented CTL priming in *Oasl1* ^−/−^ mice following mucosal HSV-2 infection

**Figure 7:**
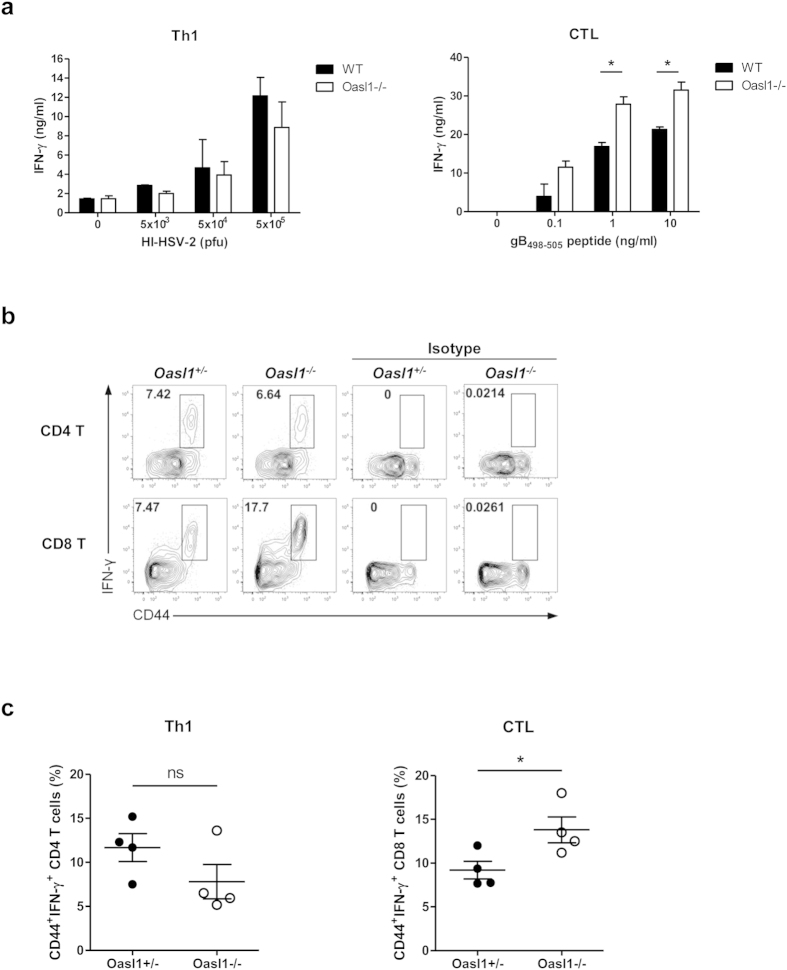
CTL priming is accelerated in *Oasl1*-deficient mice during mucosal HSV-2 infection. (**a**) WT and *Oasl1*^−/−^ mice were infected intravaginally with 10^6^ pfu of TK- HSV-2. At day 6 post-infection, CD4 and CD8 T cells isolated from draining lymph nodes were restimulated with heat-inactivated HSV-2 or gB peptide for 72 h, and IFN-γ production was measured by ELISA (n = 2 mice). Data are representative of two independent experiments. (**b,c**) *Oasl1*^+/−^ and *Oasl1*^−/−^ mice were infected intravaginally with 5000 pfu of WT HSV-2. (**b**) At day 6 post-infection, IFN-γ production from activated CD4^+^ or CD8^+^ T cells isolated from draining lymph nodes was measured by intracellular cytokine staining after stimulation with PMA and ionomycin. (**c**) Frequency of CD44^+^IFN-γ^+^ of CD4^+^ and CD8^+^ T cells was assessed (n = 4 mice). Data are representative of three independent experiments. *p < 0.05; ns, not significant. Error bars: SEM.

There have been many studies investigating the role of type I IFNs in antiviral T cell responses^6^,^24^. To determine whether the ability of *Oasl1*^−/−^ mice to induce enhanced type I IFN responses also affects the priming of virus-specific T cells, we measured IFN-γ production by CD4^+^ and CD8^+^ T cells from draining lymph nodes after mucosal HSV-2 infection. We found that the level of IFN-γ produced by CD8^+^ T cells, but not CD4^+^ T cells, was increased in *Oasl1*^−/−^ mice compared to WT mice (Fig. 7a). Consistently, the frequency of IFN-γ-producing activated (defined as CD44^+^) CD8^+^ T cells was also increased in *Oasl1*^−/−^ mice in response to mucosal HSV-2 infection (Fig. 7b,c). Collectively, these results suggest that CTL priming, but not Th1 priming, is augmented in *Oasl1*^−/−^ mice following mucosal HSV-2 infection.

## Discussion

In this study, we investigated how OASL1 affects antiviral protection against mucosal HSV-2 infection. We show that *Oasl1*^−/−^ mice exhibit better survival rates and efficiently suppressed viral replication in spite of recruitment comparable to that of WT mice of effector immune cells into the site of infection. Notably, this enhanced protection was attributed to hematopoietic cells. In this regard, we also show that BM cells from *Oasl1*^−/−^ mice produced markedly higher levels of type I IFN after stimulation with HSV-2, and that this enhanced production of type I IFN was induced in Ly6C^high^ monocytes and not by pDCs or other cell types. However, the level of type III IFN was not increased in *Oasl1*^−/−^ BM cells compared with *Oasl1*^+/−^ BM cells after stimulation with HSV-2. Furthermore, after mucosal HSV-2 infection, cytotoxic T cell responses, including IFN-γ production, are augmented in *Oasl1*^−/−^ mice compared to WT mice although the level of DC maturation is not enhanced in *Oasl1*^−/−^ mice.

The higher level of type I IFN and effective protection against viral infection observed in the present study complements our previous study in which we showed that *Oasl1*^−/−^ mice are more resistant to systemic viral infections due to enhanced production of type I IFN^15^. Notably, one important difference between these studies is the route of viral entry and, thus, the cell types participating in innate immune responses. Unlike systemic viral infection, both hematopoietic and stromal cells take part in antiviral immunity against local mucosal viral infection. Upon respiratory viral infection, airway epithelial cells rapidly recognize viral pathogens through surface-expressed or cytoplasmic pattern recognition receptors. They, in turn, produce various kinds of antiviral proteins including antimicrobial peptides, IFNs, cytokines and chemokines, promoting recruitment of immune cells and inducing adaptive immune responses^25^. Likewise, epithelial cells in the female reproductive tract, as well as hematopoietic cells, function in antiviral immunity^26^,^27^. In the case of IFN signaling, because hematopoietic cells were reported to be more important than stromal cells^21^, we hypothesized that hematopoietic cells from *Oasl1*^−/−^ mice contribute more to the enhanced production of type I IFNs and provide better protection against mucosal HSV-2 infection. Using BM chimera mice, we confirmed that hematopoietic cells from *Oasl1*^−/−^ mice are sufficient for the enhanced antiviral protective immunity (Fig. 2). Of note, how stromal compartments, including epithelial cells, contribute to the enhanced protection against mucosal HSV-2 infection observed in *Oasl1*^−/−^ mice versus WT mice remains to be investigated.

The type III IFNs (IFN-λ) are newly identified cytokines important in mucosal antiviral protection^7^,^8^. Although type III IFN binds to a different receptor complex than type I IFN, these cytokines share similar signaling pathways including the activation of IRF7^9^. In our study, *in vitro* stimulation with HSV-2 did not induce increased production of IFN-λ despite the induction of much higher levels of IFN-α and IFN-β in *Oasl1*^−/−^ BM cells compared to *Oasl1*^+/−^ BM cells (Fig. 3a–c). In the case of mucosal HSV-2 infection, the level of IFN-λ in vaginal washes from *Oasl1*^−/−^→WT mice did not decrease and was maintained until late time points post-infection; however, the amount of IFN-λ at early time points in *Oasl1*^−/−^→WT mice did not differ from that of *Oasl1*^+/−^→WT mice (Fig. 2d). This apparent discrepancy between *in vitro* and *in vivo* assays regarding the effect of OASL1 on IFN-λ production might be explained by dynamic IFN production including the effects of positive feedback. Thus, because the type I IFN receptor system also mediates positive feedback of IFN-λ expression^13^, high levels of type I IFN in the vaginal tract of *Oasl1*^−/−^→WT mice at early times after HSV-2 infection could promote the enhanced production of IFN-λ at late time points post-infection. However, we could not detect any differences in the IFN-λ production of BM cells likely due to the limited time points of stimulation.

While pDCs are known to be an important source of type I IFN in response to HSV-2 infection and antibody-mediated depletion of pDCs abrogates the expression of type I IFN^22^, recent study using CLEC4C-DTR transgenic mice to specifically deplete pDCs showed that pDCs are not the only source of type I IFNs during HSV infection^23^. To this end, we identified an additional cellular source of type I IFN after stimulation with HSV-2, Ly6C^high^ monocytes (Fig. 4b). Interestingly, Ly6C^high^ monocytes, but not pDCs, contributed to the enhanced expression of type I IFNs in *Oasl1*^−/−^ cells after stimulation with HSV-2 (Fig. 4c). This is in agreement with our previous study that showed *Oasl1*^−/−^ mouse embryonic fibroblasts and BM-derived pDCs do not express more type I IFN than do WT cells although the amount of IRF7 protein in *Oasl1*^−/−^ cells was much greater than that in WT cells^15^. Based upon the observation that pDCs showed constitutively high levels of IRF7^28^, translation of IRF7 in *Oasl1*^+/−^ pDCs was probably sufficient to produce type I IFN after stimulation with HSV-2, thus leading to no substantial differences in the expression of type I IFN between *Oasl1*^+/−^ and *Oasl1*^−/−^ cells. Taken together, in *Oasl1*^−/−^ mice diverse cell types may differentially contribute to type I IFN production in response to various viruses and routes of infection.

In addition to suppressing early viral replication, enhanced levels of type I IFN in *Oasl1*^−/−^ mice act both directly and indirectly on CD8^+^ T cells to provide further protection from viral infection. Although type I IFN was reported to act on CD4^+^ T cells to promote Th1 differentiation *in vivo*^29^,^30^, CD4^+^ T cell priming after mucosal HSV-2 infection in *Oasl1*^−/−^ mice was comparable to that in *Oasl1*^+/−^ mice in the present study (Fig. 7). Although both IFN-α and IL-12 can activate T cells to produce IFN-γ, a cytokine essential for protection against viral infection, IL-12 induces more efficient production of IFN-γ than does IFN-α^30^. Thus, these results are consistent with the observation that after stimulation with HSV-2, *Oasl1*^+/−^ and *Oasl1*^−/−^ cells showed comparable levels of IL-12p40 production (Fig. 3d). Type I IFN was also reported to enhance the function of DC by promoting their maturation including upregulation of co-stimulatory and MHC molecules^31^,^32^,^33^. In the case of HSV-2 infection, expression of co-stimulatory and MHC molecules was not significantly higher in *Oasl1*^−/−^ mice than in *Oasl1*^+/−^ mice *in vivo* and *in vitro* (Fig. 6). In this regard, it is likely that the level of type I IFNs produced in *Oasl1*^+/−^ mice was sufficient to induce DC maturation.

Consistent with our previous study, the present study demonstrates that loss of OASL1, a negative regulator of IRF7, promotes antiviral immunity against mucosal HSV-2 infection through robust production of type I IFN. These results emphasize the potential of OASL1 as an antiviral target for boosting the production of type I IFN during HSV-2 infection of the genital mucosa.

## Methods

### Mice

*Oasl1*^−/−^ mice were described in our previous study^15^. IFNβ^mob/mob^ mice have been reported previously^34^. *Oasl1*-deficient IFNβ^mob/mob^ mice were bred in-house. Specific pathogen-free C57BL/6 mice were purchased from DBL Co. Ltd, Korea. All mice were housed in a specific pathogen-free facility at KAIST. All animal experiments were performed in accordance with the guidelines and policies for rodent experimentation provided by the Institutional Animal Care and Use Committee (IACUC) of KAIST. The study protocol was approved by the IACUC of KAIST (IACUC-13-140).

### Virus and intravaginal infection

HSV-2 WT, HSV-2 TK-, and HSV-1 GFP strains provided by A. Iwasaki (Yale University, New Haven, CT) were used for all experiments. HSV-2 was propagated and titrated by a plaque assay on Vero cells. For intravaginal virus infection, mice were injected subcutaneously with medroxyprogesterone acetate (Tokyo Chemical Industry Co., Ltd.) at 2 mg/mouse in 100 μL volume 5–7 d prior to infection, swabbed with calcium-alginate, and inoculated intravaginally with 500–10000 pfu of HSV-2 WT, or with 10^6^ or 10^7^ pfu HSV-2 TK- in a 10 μL volume using a blunt-ended micropipette tip, as previously described^35^. Upon WT HSV-2 challenge, the severity of disease was scored as follows^36^: 0, no sign of disease; 1, slight genital erythema and edema; 2, moderate genital inflammation; 3, purulent genital lesions; 4, hind-limb paralysis; 5, pre-moribund. Due to humane concerns, animals were euthanized prior to reaching the moribund state.

### Vaginal viral titration and cytokine measurement

Vaginal fluids were collected on the indicated days by pipetting a volume of 50 μL of PBS in and out of the vagina 20 times. Viral titers were measured by titration of vaginal fluids on Vero cells for 72 h in duplicate as described previously^37^. The levels of IFN-α (eBioscience, San Diego, CA) and IFN-λ (R & D systems, Minneapolis, MN) in vaginal fluids were measured by ELISA according to manufacturer’s instructions.

### RNA isolation, cDNA preparation, and RT-PCR

RNA was isolated from sorted cells or total vaginal tissue through mechanical homogenization followed by use of the RNeasy Plus Mini Kit (QIAGEN), and cDNA was synthesized using the NobleZyme™ M-MLV reverse transcriptase (Noble Bio). Both were performed according to the manufacturer’s instructions. Quantitative PCR was performed using the CFX96 Real-Time PCR detection system with SYBR Green-based quantification (Bio-Rad) with the following gene-specific forward (F) and reverse (R) primers: *Oas1* F:GCCTGATCCCAGAATCTATGC and R:GAGCAACTCTAGGGCGTACTG^38^; *Irf7* F:CAGCAGCAGTCTCGGCTTGTG and R:TGACCCAGGTCCATGAGGAAGTG^20^; *Isg15* F:GGTGTCCGTGACTAACTCCAT and R: TGGAAAGGGTAA-GACCGTCCT^39^; *Ifnβ* F:GCACTGGGTGGAATGAGACTATTG and R:TTCTGAGGCATCAACTGACAGGTC ; HSV-2 gB F:CACCGCTACTCCCAGTTTATG and R: CGGTGGTCTCCATGTTGTT ; *Hprt* F:GTTGGATACAGGCCAGACTTTGTTG and R:GAGGGT-AGGCTGGCCTATTGGCT; *Gapdh* F:CACTCTTCCACCTTCGATGCC and R:CCTTGGAGGCCATGTAGGCC. Genes of interest were normalized to *Hprt* or *Gapdh* and results displayed as the fold difference relative to WT control mice.

### RT-qPCR for sorted CD4^+^ T cells

Sorted CD4^+^ T cells from vaginal tissues were lysed and one-step RT-qPCR was performed using SingleShot™ SYBR Green One-Step Kit (Bio-rad) according to the manufacturer’s instructions. The RT-qPCR reaction was performed using the CFX96 Real-Time PCR detection system (Bio-Rad) with the primers described above.

### Generation of bone marrow chimera mice

BM cells were isolated from the femurs and tibiae of mice, and single cells were prepared by passage through 70 μm cell strainers (SPL). To generate chimeric mice, recipient mice (C57BL/6) were lethally irradiated two times with 4.75 Gy of γ-irradiation 3 h apart for a total exposure of 9.5 Gy from a ^137^Cs source (Gamma Cell-1000 Irradiator; Nordion Inc, Kanata, ON, Canada). Each recipient received 5 × 10^6^ BM cells via tail vein injection. Transplanted mice were maintained on oral antibiotic in the drinking water for 3 weeks. Eight weeks post-reconstitution, chimeric mice were used for experiments.

### Stimulation of total bone marrow cells

BM cells were isolated from the femurs and tibiae of mice, and single cells were prepared by passage through 70 μm cell strainers (SPL). 5 × 10^5^ or 1 × 10^6^ BM cells were stimulated with GFP HSV-1 or TK- HSV-2 at the indicated MOIs and cultured at 37 **°**C for 18 h. Levels of IFN-α, IFN-β (eBioscience), IFN-λ (R & D systems), and IL-12p40 (BD Biosciences, San Jose, CA) from supernatants were measured by ELISA. Cells were collected and stained for flow cytometric analysis.

### Stimulation of BM-derived DCs

BM-derived DCs (BM-DCs) were generated by incubation of BM cells with 5% granulocyte-macrophage colony-stimulating factor (GM-CSF)-supplemented RPMI 1640 media containing 10% FBS (HyClone, Logan, UT) and penicillin/streptomycin (Welgene) for 5 days, as described previously^40^. 2.5 × 10^5^ BM-DCs were stimulated with TK- HSV-2 at the indicated MOIs and cultured at 37 **°**C for 18 h. Cells were collected, stained, and analyzed by flow cytometric analysis.

### Flow Cytometry

Single-cell suspensions were prepared from the iliac lymph nodes and vaginal tissue based on previously described methods, with modifications^41^. Briefly, lymph nodes were digested with Collagenase type IV (Worthington) and DNase I (Roche). Vaginas were treated with Dispase II (Roche) for 15 min and then digested with Collagenase type IV (Worthington), DNase I (Roche), and hyaluronidase (MP Biomedicals) for 45 min. Single cells were pretreated with anti-CD16/32 (2.4G2) antibody to block Fc receptors and stained with following antibodies: B220 (RA3-6B2), Ly6C (AL-21), Ly6G (1A8), CD45.2 (104), CD11c (HL3), CD11b (M1/70), MHCII (M5/114.15.2), NK 1.1 (PK136), CD8α (53-6.7), and CD86 (GL1) (BD Biosciences); CD3ε (145-2C11), CD11c (N418), and CD317 (BST2, eBio927) (eBioscience); MHCI (H-2K^b^, AF6-88.5) (BioLegend, San Diego, CA). BM cells stimulated with HSV-2 were stained with following antibodies: B220 (RA3-6B2), Ly6C (AL-21), Ly6G (1A8), CD11b (M1/70), and Siglec-F (E50-2440) (BD Biosciences); CD3ε (145-2C11), CD19 (eBio1D3), CD317 (BST2, eBio927), F4/80 (BM8), and CD49b (DX5) (eBioscience); Siglec-H (551), CD11c (N418), and MHCII (M5/114.15.2) (BioLegend). BM-DCs stimulated with HSV-2 were pretreated with anti-CD16/32 (2.4G2) antibody to block Fc receptors and stained with following antibodies: MHCII (M5/114.15.2), CD11b (M1/70), and CD86 (GL1) (BD Biosciences); CD11c (N418) (BioLegend). Cells were gated on the basis of forward and side scatter properties, and live cells were gated on the basis of propidium iodide (PI) or 4’,6-diamidino-2-phenylindole (DAPI) exclusion. All samples were acquired on an LSR Fortessa or LSRII (BD Biosciences). Leukocytes from iliac lymph nodes of infected mice were cultured in the presence of 50 ng/mL phorbol myristate (PMA) (Sigma-Aldrich, St. Louis, MO) and 500 ng/mL ionomycin (Sigma-Aldrich) for 5 h, and 2 μM GolgiStop (BD Biosciences) was added for the final 2 h. Cells were stained with Fixable Viability Stain 450 (BD Biosciences) in order to exclude dead cells. Cells were then surface stained with CD3ε (145-2C11), CD4 (GK1.5) (eBioscience), CD8α (53-6.7), and CD44 (IM7) (BD Biosciences) and were fixed and permeabilized using a Cytofix/Cytoperm kit (BD Biosciences) according to manufacturer’s instructions. For intracellular cytokine staining, APC-labeled anti-mouse IFN-γ Ab (XMG1.2; BD Biosciences) was used. All data were analyzed with FlowJo (Treestar).

### Vaginal/BM monocyte and vaginal CD4^+^ T cell sorting

Single-cell suspensions were prepared from the bone marrow of uninfected WT mice and intravaginal WT HSV-2 infected vaginal tissues of *Oasl1*^+/−^ or *Oasl1*^−/−^ mice. To obtain FACS-sorted Ly6C^high^ monocytes and CD4^+^ T cells, cells were stained with Ly6C (AL-21), Ly6G (1A8), CD11b (M1/70), and CD45.2 (104) (BD Biosciences); CD3ε (145-2C11) and CD4 (GK1.5) (Tonbo biosciences) after incubating for 15 min on ice in the presence of anti-CD16/32 (2.4G2, Tonbo biosciences) antibody to block Fc receptors. Live cells were gated on the basis of DAPI exclusion. Stained cells were sorted by FACS Aria (BD Biosciences). Sorted vaginal Ly6C^high^ monocytes (CD45.2^+^Ly6G^-^Ly6C^high^CD11b^+^) and CD4^+^ T cells (CD45.2^+^CD3ε^+^CD4^+^) were more than 90% pure as assessed by post-sort analyses.

### CD4^+^ and CD8^+^ T cell responses

HSV-specific T cell responses were analyzed as previously described^42^. At day 6 post-infection, CD4^+^ and CD8^+^ T cells were isolated from the draining lymph nodes of mice infected intravaginally with 10^6^ pfu of TK- HSV-2 using anti-CD4- or anti-CD8-conjugated microbeads (Miltenyi Biotech) according to the manufacturer’s instructions. 1 × 10^5^ CD4^+^ and CD8^+^ T cells were co-cultured with 2 × 10^5^ splenocytes as antigen presenting cells and restimulated with various amounts of heat-inactivated HSV-2 or HSV glycoprotein B (gB) peptide (SSIEFARL) at 37 **°**C for 72 h. IFN-γ production in supernatants was measured by ELISA.

### Statistical analysis

Data are expressed as mean ± standard error of means (SEM). Differences between groups at individual time points were analyzed using the unpaired, two-tailed Student’s *t*-test. Disease scores were analyzed by two-way ANOVA test. Differences in survival were evaluated by the Log-Rank test. Statistical analysis was performed using GraphPad Prism 5.01 software (GraphPad Software, San Diego, CA). Differences were considered significant when the P value was <0.05 and are indicated as follows: *p < 0.05; **p < 0.01; and ***p < 0.001.

## Additional Information

**How to cite this article**: Oh, J. E. *et al.* OASL1 deficiency promotes antiviral protection against genital herpes simplex virus type 2 infection by enhancing type I interferon production. *Sci. Rep.* **6**, 19089; doi: 10.1038/srep19089 (2016).

## Supplementary Material

Supplementary Information
