# Supplementary material for: OASL1 deficiency promotes antiviral protection against genital herpes simplex virus type 2 infection by enhancing type I interferon production
Source: Sci Rep. 2016 Jan 11;6:19089. doi: 10.1038/srep19089 (PMC4707503; doi:10.1038/srep19089)
Supplement: Supplementary Information [file srep19089-s1.pdf]

Supplementary information

OASL1 deficiency promotes antiviral protection against genital herpes simplex virus type 2 infection by enhancing type I interferon production

Ji Eun Oh[1,+], Myeong Sup Lee[2,+], Young-Joon Kim[3,4], Heung Kyu Lee[1*]

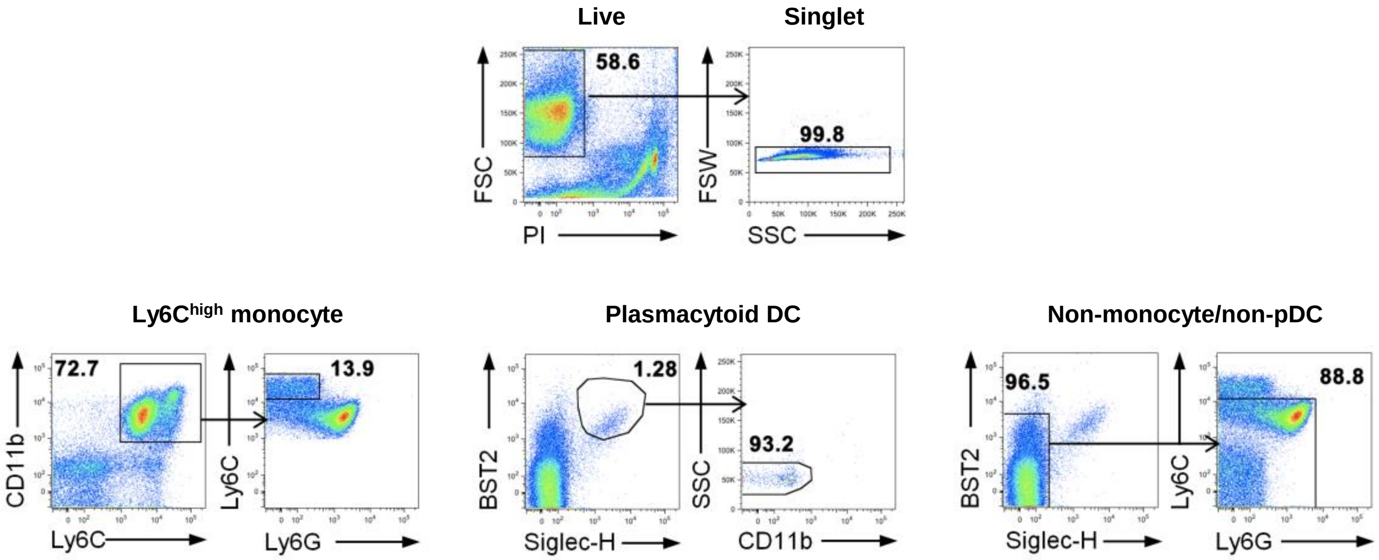

Supplementary Figure 1. Gating strategies for analysis of Ly6C^{high} monocytes, plasmacytoid DCs (pDCs), and non-monocyte/non-pDC BM cells. Live cells were gated based on forward scatter and PI exclusion. Ly6C^{high} monocytes were defined as Ly6C^{high}CD11b^{+}Ly6G^{-} cells and pDC as Siglec-H^{+}BST2^{+}CD11b^{-} cells.

a

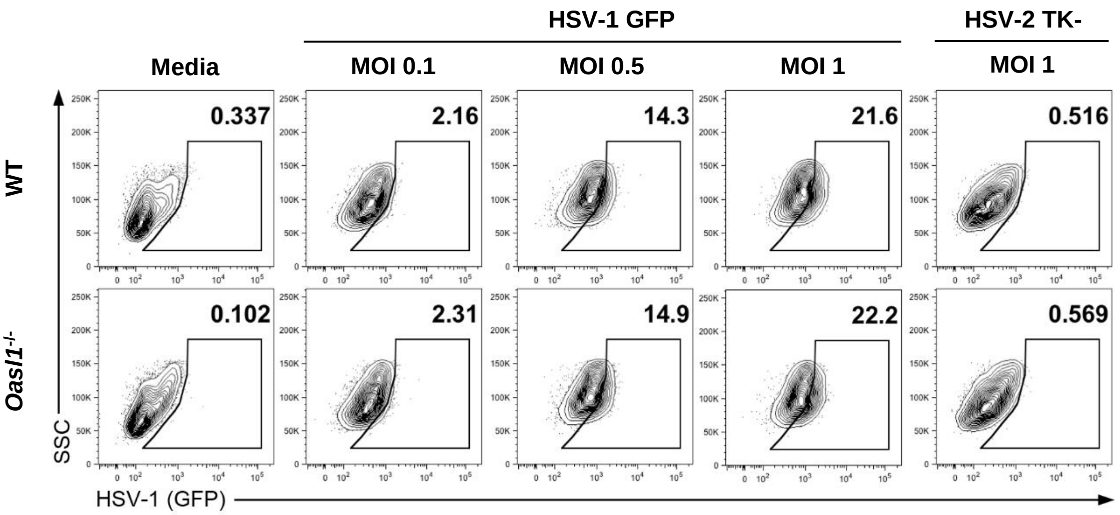

b

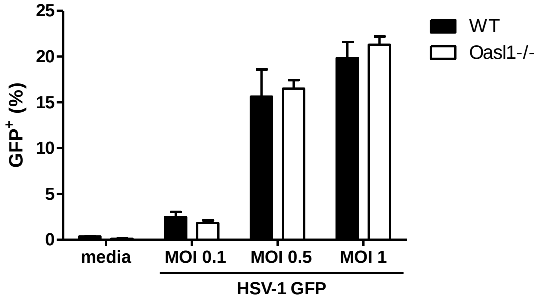

Supplementary Figure 2. HSV directly infects bone marrow monocytes *in vitro*. BM cells from WT and *Oasl1*$^{-/-}$ mice were stimulated with GFP HSV-1 at the indicated MOIs for 18 h. (a) GFP expression in Ly6C^{high} monocytes was assessed using flow cytometry. Numbers indicate the percentage of cells with GFP expression. BM cells stimulated with TK- HSV-2 were used as controls for GFP expression. (b) Bar graphs show the percentage of GFP expression in (a) (n=3). Data are representative of three independent experiments.

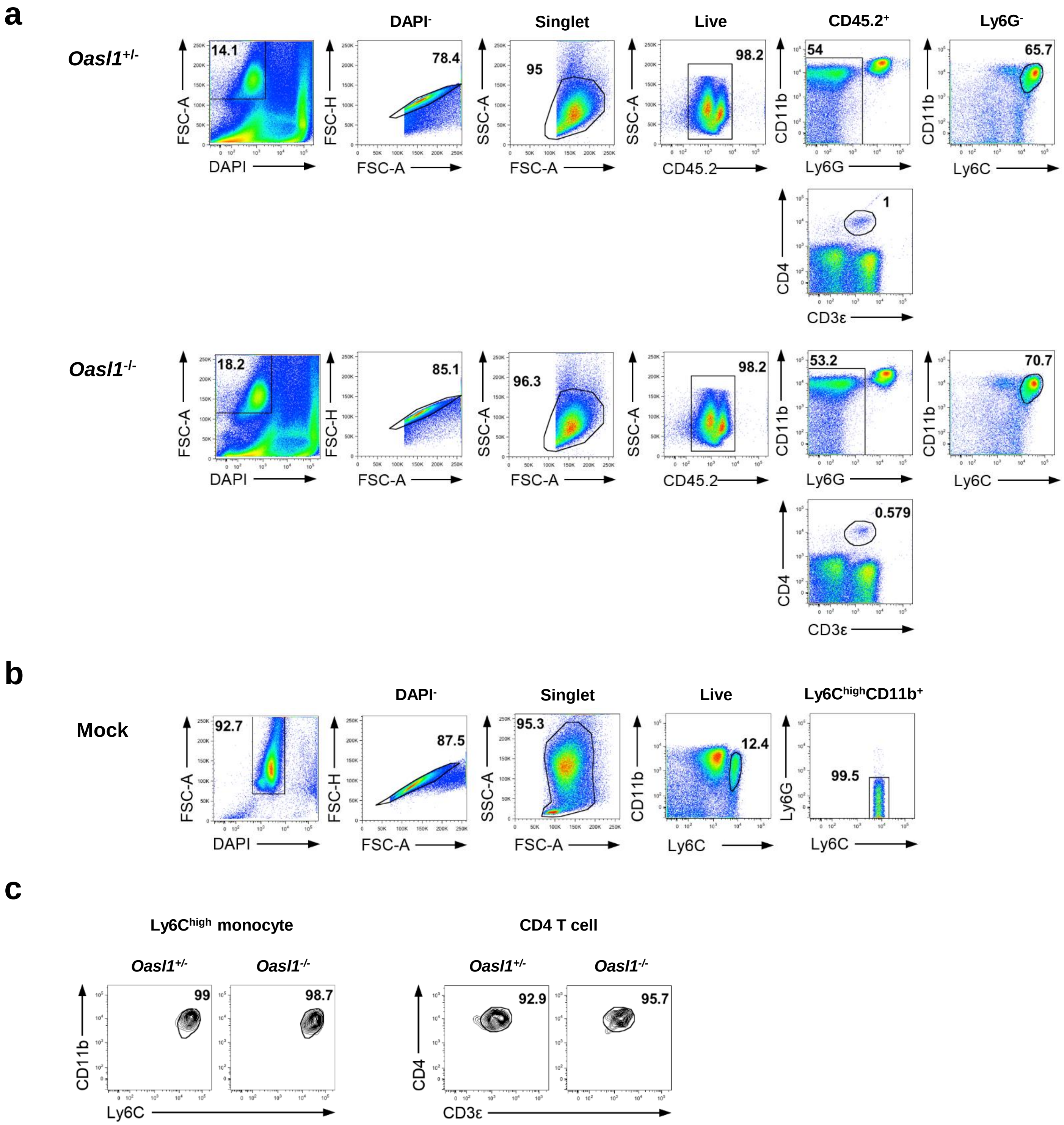

Supplementary Figure 3. Gating strategies for sorting of Ly6C^{high} monocytes and CD4^{+} T cells. (a) Gating strategies for Ly6C^{high} monocytes and CD4^{+} T cells from the vaginal tissue of intravaginal WT HSV-2 infected *Oasl1*$^{+/-}$ and *Oasl1*$^{-/-}$ mice. Live cells were gated based on forward and side scatter and DAPI exclusion. The CD45.2^{+} cells were further gated on Ly6C^{high} monocytes (Ly6G^{-}Ly6C^{high}CD11b^{+}) and CD4^{+} T cells (CD3ε^{+}CD4^{+}). (b) Gating strategy of Ly6C^{high} monocytes from BM cells of uninfected WT mice. Live cells were gated based on forward and side scatter and DAPI exclusion. Ly6C^{high} monocytes were defined as Ly6C^{high}CD11b^{+}Ly6G^{-} cells. (c) Purity of Ly6C^{high} monocytes and CD4^{+} T cells sorted from the vaginal tissue of intravaginal WT HSV-2 infected *Oasl1*$^{+/-}$ and *Oasl1*$^{-/-}$ mice was analyzed by flow cytometry.
